# Molecular phylogeny and morphology reveal a new epiphytic species of *Habenaria* (Orchidaceae; Orchideae; Orchidinae) from Nepal

**DOI:** 10.1371/journal.pone.0223355

**Published:** 2019-10-23

**Authors:** Bhakta Bahadur Raskoti, Rita Ale

**Affiliations:** 1 Nature Research and Conservation Initiatives, Pokharathok, Arghakhanchi, Nepal; 2 Nepal Bioscience Research Laboratory, Banasthali, Kathmandu, Nepal; University of Sao Paulo, BRAZIL

## Abstract

*Habenaria* is almost cosmopolitan in distribution having predominantly terrestrial orchids, however; a remarkable epiphytic species with some unique morphological characters was collected from Nepal. We conducted a molecular phylogeny of this unusual *Habenaria* species using nuclear (ITS) and chloroplast (*matK*, *rbcl*) DNA sequence regions to infer its systematic position. Our molecular analyses and morphological treatment recognized this newly collected plant as an undescribed species. This species is described here which is closely related to *Habenaria plurifoliata* but can be distinguished by having its multiple growing callus-shaped tuber, smaller stature with short stem, longer and wider oblanceolate leaves, peduncle with a foliaceous bract and only one sterile bract, lateral sepals oblong, obtuse, petal apex obtuse, lateral lobes of lip spreading upwards, spur not exceeding the ovary and pedicel.

## Introduction

*Habenaria* Willd. (Orchidinae, Orchidoideae, Orchidaceae) is a large genus having more than 891 species [1]. The vast majority species of *Habenaria* are terrestrial orchids and nearly cosmopolitan, occurring in the tropical, subtropical, temperate and alpine regions [2–5]. *Habenaria* as currently delimited is characterized by having undivided tuber, spurred lip, long column, U-shaped wide anther connective, long caudicle, naked viscidium, long and free stigma drawn out at the entrance of spur [2,3]. Molecular phylogeny performed by different authors that included *Habenaria* and its alliance (particularly *Herminium* L., *Hsenhsua* X.H. Jin, Schuit. et W.T. Jin, *Bonatea* Willd., *Diplomeris* D. Don, *Gennaria* Parl., *Peristylus* Blume) indicated that *Habenaria*, in the broad sense, is not monophyletic [6, 7]. Despite of past and recent progress on phylogeny of *Habenaria* and its alliance [6, 7, 8, 9], problems remain to be addressed to solve relationships mainly between Old World and New World species [6, 7].

New descriptions of *Habenaria* species from both Old World and New World are still going on which is evident from its series of recent studies [10–20]. In the same context, a remarkable epiphytic species of *Habenaria* was collected from Nepal Himalaya by the first author. Several morphological characters of this species did not completely match with other described species of *Habenaria*. Hence, we performed molecular phylogeny of *Habenaria* and its alliance using nuclear ribosomal ITS and plastid *matK*, *rbcl* sequences in order to ascertain systematic position of the newly collected species.

## Materials and methods

### Ethics statement

The collecting location reported in this work is not protected area by any law. In addition, the plant species collected here are currently neither endangered nor protected. No specific permits are required for the present study.

### Nomenclature

The electronic version of this article in Portable Document Format (PDF) in a work with an ISSN and ISBN will represent a published work according to the International Code of Nomenclature for algae, fungi, and plants, and hence the new names contained in the electronic publication of PLoS article are effectively published under that Code from the electronic edition alone, so there is no longer any need to provide printed copies.

In addition, new name contained in this work has been submitted to The International Plant Names Index (IPNI), from where they will be made available to the Global Names Index (http://gni.globalnames.org/). The IPNI LSIDs can be resolved and the associated information viewed through any standard web browser by appending the LSID contained in this publication to the prefix http://ipni.org/. The online version of this work is archived and available from the following digital repositories: PubMed Central and LOCKSS.

### Morphological observations

Vegetative and reproductive characters were studied based on fresh and preserved specimens. Characters such as tuber, stem, phyllotaxy, leaf shape, bracts, sepals, petals, lip and column were carefully examined and measured. The characters were compared with allied species deposited in different herbaria, and information published in different literatures. The plants were dried and herbarium specimens were prepared following the standard procedure and deposited them in the National Herbarium and Plant Laboratories (KATH), Godawari, Lalitpur, Nepal.

### Taxon sampling

In total, 195 sequences of 189 species were used in the phylogenetic study. We downloaded 193 sequences of 188 species from GenBank. Additionally, we sequenced two samples of the newly collected taxon for the present analyses. The ingroup taxa included a total of 191 sequences of 185 species, representing all of the major clades in the *Habenaria* and its alliance [6, 7, 8, 9]. Three representative species of *Disa* P.J. Bergius and one species of *Goodyera* R. Br. were selected as outgroups following previous phylogenetic studies [6, 7, 8]. We added 41 additional samples of *Habenaria* species than in the past phylogenetic study [7]. A list of sampled species, GenBank accession numbers for all sequences and voucher information are provided in S1 Appendix.

### DNA extraction and marker selection

DNA extraction was carried out from fresh leaf tissue using the modified CTAB protocol [21]. Amplification of DNA regions was performed using a standard polymerase chain reaction (PCR). The sequencing reactions were performed using the ABI Prism Bigdye Terminator Cycle Sequencing Kit (Applied Biosystems, ABI) following the reference Raskoti et al. [22]. We selected nucleotide sequences (ITS), and plastid (*matK* and *rbcl*), which have exhibited their efficacy for understanding phylogenetic relationships at various taxonomic levels within the Orchidinae and other members in Orchidaceae [6, 7, 9, 22–25].

### Sequence alignment and phylogenetic reconstruction

DNA sequences were initially aligned with the help of CLUSTAL X version 1.83 [26] with minor subsequent manual adjustment using the software BioEdit verion 5.0.9 [27]. To evaluate congruence between different DNA regions, we analyzed each nuclear (ITS) and chloroplast (*matK* and *rbcl*) dataset separately to see if they produce an identical topology. Homogeneity between the nuclear (ITS) and plastid datasets (*matK*, *rbcL*) was tested using the incongruence length difference (ILD) test [28] with 500 replicates of the heuristic search applied in PAUP v.4.0b10 [29]. The 1% level of significance was chosen according to Darlu & Lecointre [30]. The ILD value in this study was 0.33, suggesting congruence between the two regions. We therefore analyzed nuclear and plastid datasets in combination. Phylogenetic analyses were carried out using maximum parsimony (MP) as implemented in PAUP* [29]. MP analyses were conducted using heuristic searches with 1,000 replicates of random taxon addition, and tree rearrangements using tree bisection-reconnection (TBR) branch swapping to obtain the parsimonious trees. All characters were treated as unordered and had equal weights, gaps considered as missing data. The node supports for clades were assessed by bootstrap (BS_MP_) analyses, which were performed using 1,000 replicates with ten random taxon additions and heuristic options [31].

For the Bayesian inference (BI) analysis, posterior probabilities (PP) were conducted in MrBayes version 3.2.3 [32], implemented in CIPRES Science Gateway [33]. The best-fitting model of sequence evolution in BI analyses was chosen for each marker using ModelTest 3.7 [34] under the Akaike Information Criterion (AIC) in conjugation with PAUP* [29]. Evolutionary models that best fitted the ITS, *matK* and *rbcl* were GTR+ I+ G (nst = 6, rates = invagamma). Two separate runs, each with four-Markov-Chain Monte Carlo (MCMC) analyses were performed, starting with a random tree, proceeding for 7,000,000 generations (for combined dataset), and sampling one tree every 1000 generations. Convergence between the runs was achieved after 2,36,000 generations which was evaluated using the average standard deviation of the split frequencies (<0.01). Majority rule (> 50%) consensus trees were reconstructed after removing the ‘burn-in phase’ samples (the first 25% of the sample trees).

## Results

**Fig 1:**
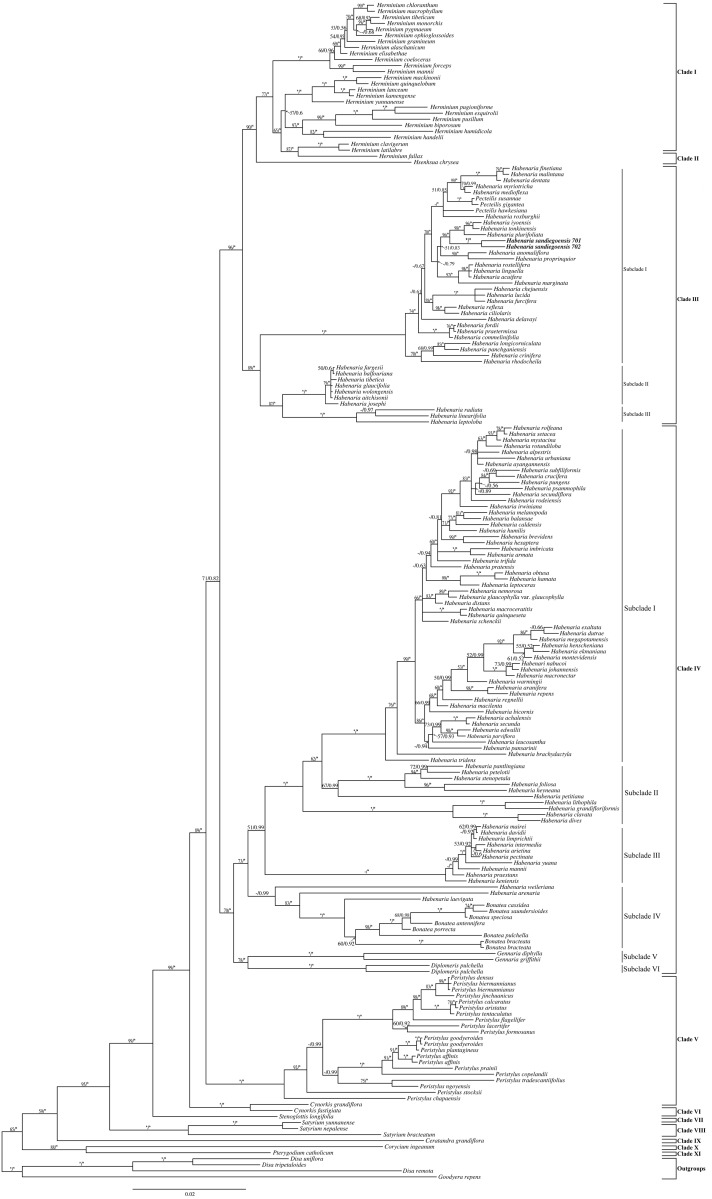
Bayesian inference tree of *Habenaria* alliance based on the combined nuclear (ITS), and plastid (*matK*, *rbcl*) markers. Numbers above branches indicate bootstrap percentages (BS_MP_) for MP analysis and posterior probabilities (PP) for BI analysis respectively. Asterisk (*) indicates BS_MP_ = 100% and PP = 1.00, a dash (−) indicates support at a node < 50%.

The length of aligned matrix of nrITS was 853 bp, of which 473 (42%) were parsimony-informative. The aligned *matK* matrix consisted of 1695 bp, of which parsimony informative characters were 490 (28%). Similarly, *rbcl* matrix was 1254 bp of which 101 characters were parsimony informative. The combined chloroplast data matrix comprised 2949 characters of which 591 characters were parsimony informative. The combined (nuclear and chloroplast) data matrix consisted of 3802 characters of which 435 were variable and 1064 (28%) were parsimony informative. There was no significant difference in topologies of individual marker and also between nrITS and chloroplast (supplementary S1, S2, S3, S4 and S5 Figs); however, they were at different levels of resolution. For the combined data, the heuristic search found 5778 MPTs with a length of 11085 steps, consistency index = 0.41 and retention index = 0.81. The topologies from the MP and BI analysis were congruent. The BI tree based on combined dataset is shown for the discussion of phylogenetic relationships (Fig 1).

The phylogenetic tree inferred from combined dataset (Fig 1), revealed that some Asian clade of Old World *Habenaria* including deeply nested *Pecteilis* (clade III) is sister to *Herminium* + *Hsenhsua* (clade I + II) with strong support (BS_MP_ = 90%, PP = 1.00). Clade III further divided into well-supported three subclades. Subclade I includes 32 species mainly from tropical, subtropical and temperate habitats. Subclade II comprises 7 species of *Habenaria* from alpine habitat whereas subclade III includes 3 species of *Habenaria* from tropical and subtropical habitat. Clade IV (sister of clade I+II+ III with support BS_MP_ = 96%, PP = 1.00), having 6 subclades, includes usually New World *Habenaria* intermixed with 10 species from Asian Old World *Habenaria* (i.e. *H*. *stenopetala*, *H*. *pantlingiana*, *Habenaria petelotii* in subclade II and *H*. *intermedia*, *H*. *pectinata*, *H*. *mairei*, *H*. *davidii*, *H*. *limprichtii*, *H*. *arietina* in subclade III). *Bonatea*, *Gennaria* and *Diplomeris* are nested in New World *Habenaria* (Clade IV/ Subclade IV, V, VI). *Peristylus* has been resolved as sister to subclade formed by clades I, II, III and IV with moderate support (BS_MP_ = 71%, PP = 0.82).

Our molecular results based on nuclear (ITS) and chloroplast (*matK*, *rbcl*) DNA sequence data indicated that newly sampled taxon nested within Clade III (Fig 1). This species form a separate lineage within Clade III/Sublcade 1 (BS_MP_ = 86%, PP = 1.00), which is strongly supported as sister to *H*. *plurifoliata* and its allies (BS_MP_ = 98%, PP = 1.00).

The major morphological similarities within the clade belonging to newly sampled species and allied taxa consist of basal clustered leaves (nearly rosette phyllotaxy and number of leaves usually more than two) and filiform lateral lobes of lip. Although, some distantly related taxa such as *Habanaria josephi* and its allies also have basal leaves, but their number of leaves always one or two, oppositely arranged and flat on the ground.

### Taxonomic treatment

**Fig 2:**
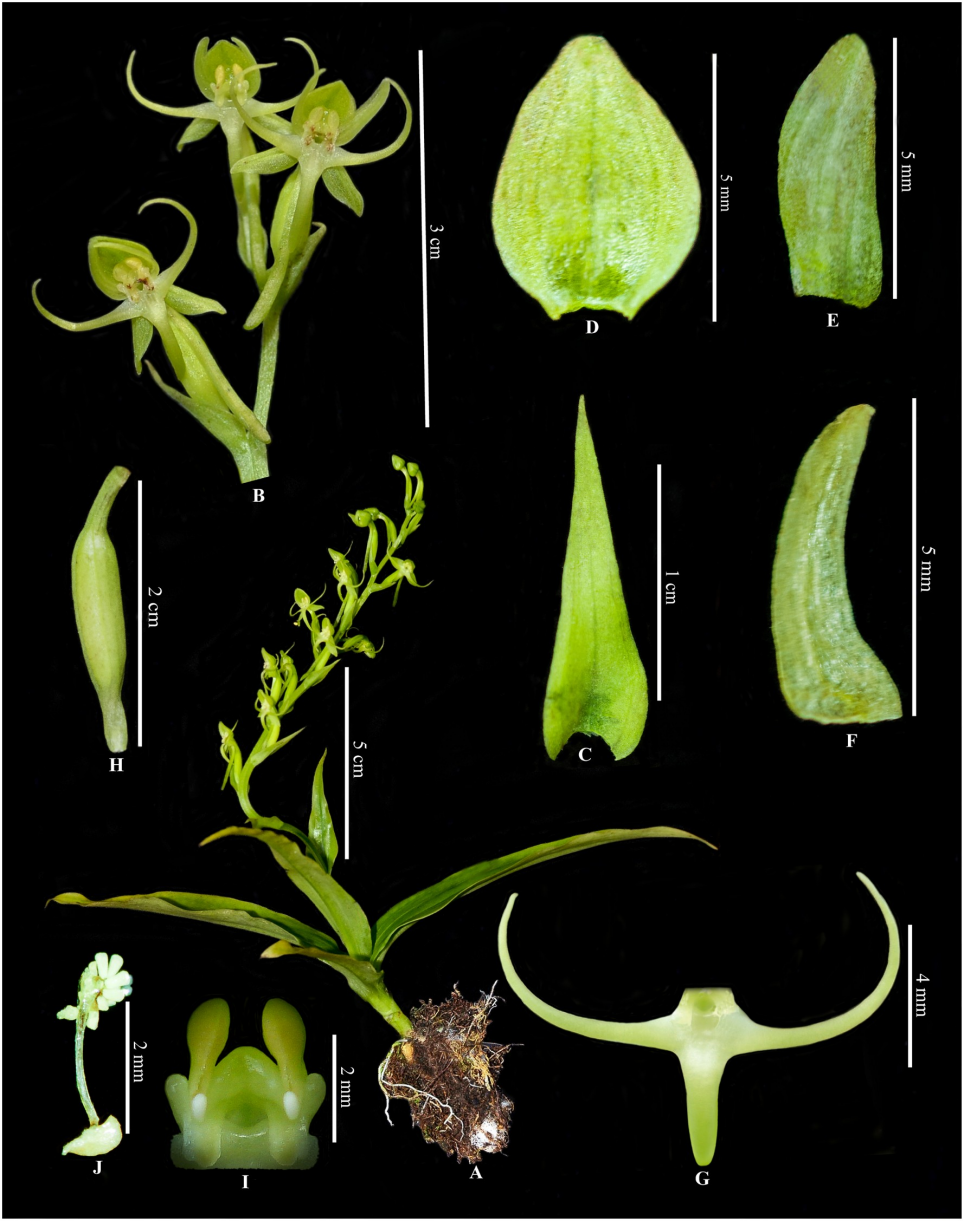
Habenaria sandiegoensis. A, Entire plant; B, Flowers with bract, pedicel and ovary; C, Floral bract; D, Dorsal sepal; E, Lateral sepal; F, Petal; G, Lip; H, Ovary and pedicel; I, Column; J, Pollinia (dissected from type specimen and all photographs by first author).

**Fig 3:**
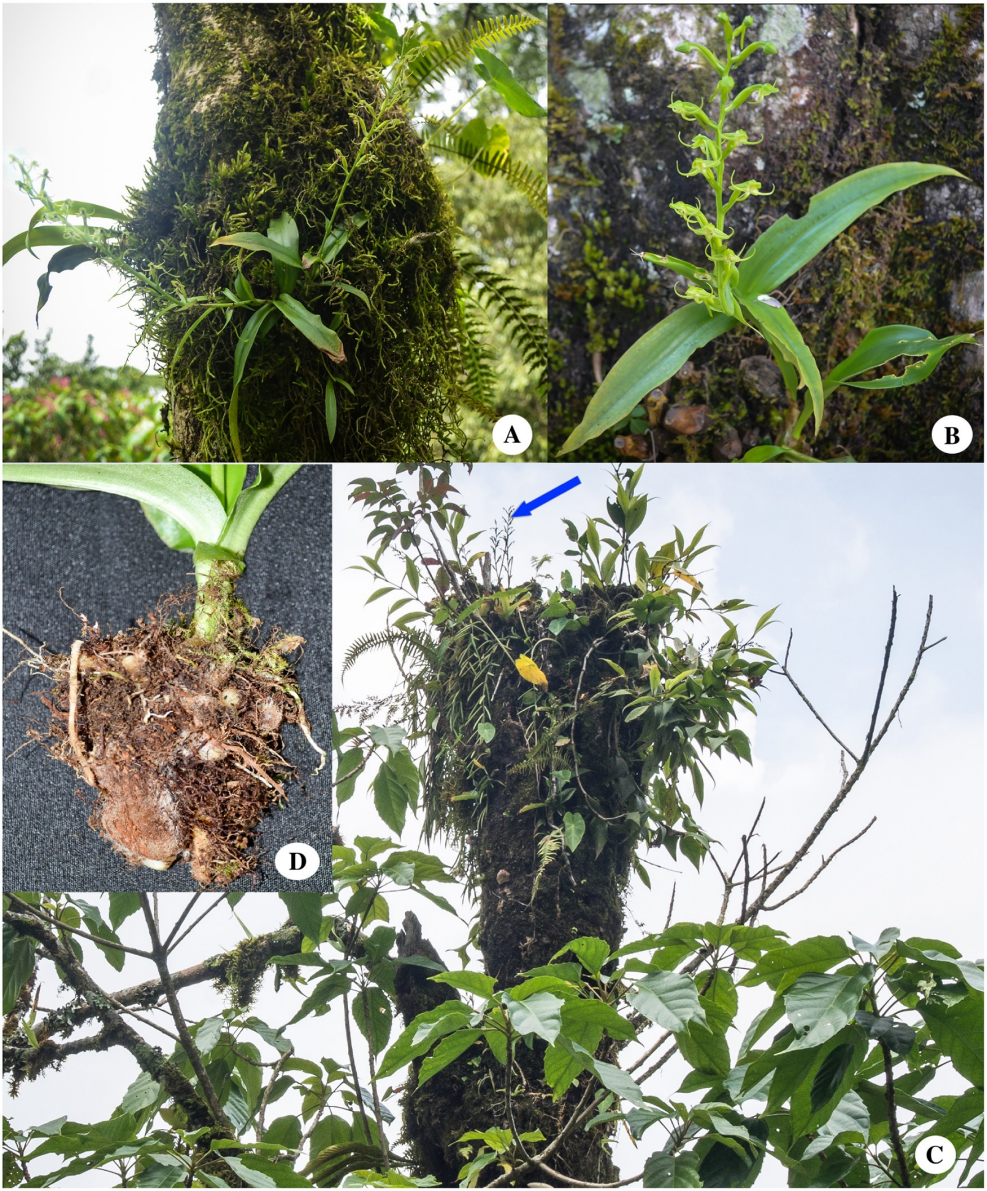
A, B, C, showing habitat and D, multiple growing callus like tubers of *Habenaria sandiegoensis*.

*Habenaria sandiegoensis* Raskoti, sp. nov. [urn:lsid:ipni.org:names: 77201935–1] (Figs 2 and 3).

Type:—NEPAL. Province 1 (Mechi Zone), Ilam district, Kutidanda, mixed deciduous forest, at 1600 m, 23 July 2010, *Bhakta B*. *Raskoti 701* (holotype: KATH).

### Diagnosis

*Habenaria sandiegoensis* is close to *Habenerai plurifoliata* Tang & F. T. Wang, but the former differs by having its epiphytic habit, multiple growing callus-shaped rhizome like tuber, smaller stature with shorter stem, more wider and longer oblanceolate leaves, peduncle with a foliaceous bract, and only one sterile bract, lateral sepals oblong, obtuse, petals apex obtuse lateral lobes of lip spreading upwards (vs. right angles to mid-lobe), spur not exceeding the ovary and pedicel.

### Description

Epiphytic herb, up to 15–25 cm tall. Tuber undivided, multiple growing, rhizome like, callus-shaped in general outline, single tuber cylindric-oblong, fleshy, partly covered by dense white soft hairs (woolly), apex with few slender roots; roots creeping, exposed in air, sometime branched near apex. Stem erect, stout, 1–2 cm long, with 3–5 leaves near base, a foliaceous bract and a sterile bract above (or absent of sterile bract). Leaves 3–5, basal clustered, base contracted into amplexicaul sheath, blade oblong-lanceolate to broadly oblanceolate, herbaceous, margin entire, apex acute, 5–12 × 1.5–2 cm. Inflorescence terminal, racemose, 12–20 cm long; peduncle cylindrical, foliaceous bract linear-lanceolate, 3–4.5 × 0.5–1 cm, apex acuminate, sterile bract absent or only one if present, ovate-lanceolate, 1.5–2.5 cm, apex acuminate; rachis 8–12 cm with 7–15 flowered; floral bracts greenish, lanceolate, shorter than ovary and pedicel, apex acuminate, 7–15 × 3–4 mm; ovary erect, twisted, cylindric-fusiform, ridged and grooved, greenish, including pedicel 1–1.5 cm, apex beaked. Flowers spreading, ca. 1.5 cm across, light-green. Dorsal sepal forming a hood with petals, erect, ovate, concave, ca. 5 × 3 mm, 3-veined, apex obtuse; lateral sepals reflexed, concave, obliquely oblong, ca. 5 × 2 mm, 3-veined, apex obtuse. Petals falcate, linear-lanceolate, ca. 5 × 1 mm, 1-veined, apex obtuse. Lip deeply 3-lobed from base; lateral lobes spreading, curved upward, filiform, 11–12 mm long; mid-lobe linear, ca. 7 × 1.2 mm, wider than lateral lobes, apex obtuse; spur pendulous, cylindrical, ca. 1–1.5 cm long, equal or shorter than ovary and pedicel (rarely an apical flower has spur minutely longer than ovary and pedicel), slender, slightly thickened toward end, apex obtuse. Column erect, 2 mm long, anther loculi erect, parallel, ca. 1 mm long, pollinia obvoid, granular-farinaceous, sectile, caudicles long, viscidia discoid; auricles 2, usually prominent, placed laterally at base of the anther; anther connective wide; rostellum lobe broad, stigma stalks short, thick.

### Phenology

Flowering and fruiting occur from July to September.

### Distribution and habitat

*Habenaria sandiegoensis* is epiphytic (Fig 3A, 3B and 3C) in moss-covered medium to large size tree trunk and main branches (from diameter of breast height to about 15 m maximum height), forming more or less scattered colonies. It grows in cloudy and moist areas, at elevation of around 1600 m in the mixed deciduous forest of Nepal Himalaya. Habitat of *Habenaria sandiegoensis* is cloudy forest, which is covered by dense fog nearly throughout the year and receives very low light intensity. So, the major cause of growing *Habenaria sandiegoensis* as an epiphytic habit could be due to competition for light. Interestingly, *Habenaria sandiegoensis* has creeping roots that are exposed in air and its tubers are multiple growing in the majority of population, which look somewhat callus-shaped and rhizome like (in general outline) and covered by dense woolly hairs (Fig 3D), the character similar to some epiphytic ferns such as *Phlebodium aureum*.

### Etymology

The epithet *sandiegoensis* derived from the name ‘San Diego County Orchid Society’ for its contribution in wild orchid conservation.

### Conservation status

*Habenaria sandiegoensis* is endemic to Nepal Himalaya, and its distribution is limited to only one population within an area of ca. 1 km^2^. In 2010, about 75 individual plants were first observed. Among them, two plants were collected for herbarium specimens. During the field works in 2013 to 2014, ca. 63 individual plants were observed at the same location, whereas, in 2016 and 2018, its number further reduced to ca. 57 and ca. 51 individuals respectively. *H*. *sandiegoensis* is restricted within a single locality and its population trend is decreasing due to deforestation of its host trees. Furthermore, habitat of type locality is highly threatened due to forest encroachment, over exploitation for firewood and fodder etc. Under these conditions, *H*. *sandiegoensis* should be evaluated as Endangered [E] based on IUCN Red List Categories and Criteria [35].

### Species recognition

*Habenaria sandiegoensis* is distinctive among its closely related taxa being the only species having epiphytic habit in the group. It is close to *Habenaria plurifoliata* (a Chinese species from Guangxi and Yunnan province), with which it shares basal clustered leaves (rosette phyllotaxy) and filiform lateral lobes of lip, but the new species can be distinguished from the later by having multiple growing callus-shaped tuber, smaller stature with short stem, leaves 3–5, oblanceolate (vs. number of leaves 6–10, linear-lanceolate), lateral lobes of lip spreading upwards, spur equal or shorter than ovary and pedicel (in very rare case, spur of an apical flower equals or slightly exceeds the ovary and pedicel). In morphology, *H*. *iyoyensis* is somewhat similar to *H*. *sandiegoensis*, but according to Chen et al. [4], former has several sterile bracts, spur longer than ovary and pedicel, lateral lobes of lip usually not spreading upward. In molecular analyses too *H*. *iyoyensis* and *H*. *sandiegoensis* were revealed different species.

*Habenaria sandiegoensis* is epiphytic in habit and has clustered leaves; in phylogenetic analyses it is clearly positioned within the terrestrial clade having taxa with clustered leaves and filiform lateral lobes. However, in the floral morphology such as shape of lateral sepal, petal, lip and column, *H*. *sandiegoensis* looks more close to *Habenaria propinquior* but later has opposite leaves and white flowers.

## Discussion

The relationships in the resulting cladograms of *Habenaria* (Fig 1), generally agree with those recovered in the previous phylogenetic analyses [6, 7, 8, 9, 22]. *Herminium* and *Hsenhsua* (clade I + II) are nested within Asian clade of Old World *Habenaria* (Clade III), letting the last not monophyletic. Clade IV/Subclade I contains the type species of *Habenaria* (*Habenaria macroceratistis* Willd), which is a Neotropical species with other species of *Habenaria* from Americas.

A monophyletic genus *Peristylus* is isolated as sister to super clade formed by clades I, II, III and IV with moderate support which is different from previous analyses where it was nested in *Habenaria* [6, 7]. This genus is usually distributed in tropical Asia; however, few species grow in subtropical to temperate climate of Asia (for example, *P*. *biermannianus*, *P*. *densus*, *P*. *calcaratus* etc.). *Peristylus* consists of 3-lobed lip often with globose spur; stigma lobes are adnate to the base of the lip.

Based on nuclear (ITS) and chloroplast (*matK*, *rbcl*) markers, phylogenetic analyses revealed a separate lineage of newly sampled species (Fig 1, Clade III/Subclade I) with BS_MP_ = 86%, PP = 1.00, which is strongly supported as sister to *H*. *plurifoliata* and its allies (BS_MP_ = 98%, PP = 1.00). Taxa belonging to newly sampled species and their closely related clade (*Habenaria tonkinensis*, *H*. *iyoensis*, *H*. *plurifoliata*) share very similar gross morphology and cannot be easily identified based on morphological traits. Although, our phylogenetic analyses do not include all members of Old World *Habenaria* (particularly in the Clade III), our sample is sufficient for the clade where newly sampled taxon nested. In our thorough investigation of the literatures, only two species (i.e. *H*. *vanoverberghii* Ames and *H*. *rosulata* Ames) endemic to Philippines could not be included in this phylogenetic analysis. While scrutinizing their morphological characters, these two species may nest within the clade having newly sampled taxa and its allies. We examined all related literatures including type protologue of *H*. *vanoverberghii* as well as *H*. *rosulata* and found that these two species are quite different from our plant by having terrestrial habitat, leaves narrower and shorter, linear to lanceolate in shape (vs. oblong-lanceolate), absence of foliaceous bracts, but presence of several sterile bracts (vs. absence of sterile bract). Similarly, other differences in floral characters are dorsal sepal lanceolate, acute (vs. ovate, obtuse), lateral sepals lanceolate, acute (vs. oblong, obtuse), petal acute (vs. obtuse), lip spur longer than ovary. Moreover, due to large disjunction in geographical regions between Nepal and Philippines, there are minimum possibilities of these two species being same to our plant. Hence, our newly sampled taxon is distinct from these two species.

## Supporting information

S1 Fig Strict consensus tree generated from combined dataset (ITS, *matK* and *rbcl*). Numbers above branches indicate bootstrap percentages for MP analysis. Asterisk (*) indicates bootstrap = 100%, support values < 50% are not shown.
(PDF)

S2 Fig Strict consensus tree generated from combined chloroplast dataset (*matK* and *rbcl*). Numbers above branches indicate bootstrap percentages for MP analysis. Asterisk (*) indicates bootstrap = 100%, support values < 50% are not shown.
(PDF)

S3 Fig Strict consensus tree generated from ITS. Numbers above branches indicate bootstrap percentages for MP analysis. Asterisk (*) indicates bootstrap = 100%, support values < 50% are not shown.
(PDF)

S4 Fig Strict consensus tree generated from *matK*. Numbers above branches indicate bootstrap percentages for MP analysis. Asterisk (*) indicates bootstrap = 100%, support values < 50% are not shown.
(PDF)

S5 Fig Strict consensus tree generated from *rbcl*. Numbers above branches indicate bootstrap percentages for MP analysis. Asterisk (*) indicates bootstrap = 100%, support values < 50% are not shown.
(PDF)

S1 Appendix Taxa analyzed, voucher information, and GenBank accession numbers for the DNA sequences. (*) Indicate sequences generated in this study.
(DOC)
