# Supplementary material for: Molecular phylogeny and morphology reveal a new epiphytic species of Habenaria (Orchidaceae; Orchideae; Orchidinae) from Nepal
Source: PLoS One. 2019 Oct 23;14(10):e0223355. doi: 10.1371/journal.pone.0223355 (PMC6808328; doi:10.1371/journal.pone.0223355)

S1 Fig. Strict consensus tree generated from combined dataset (ITS, *matK* and *rbcl*).
Numbers above branches indicate bootstrap percentages for MP analysis.
Asterisk (*) indicates bootstrap = 100%, support values < 50% are not shown.

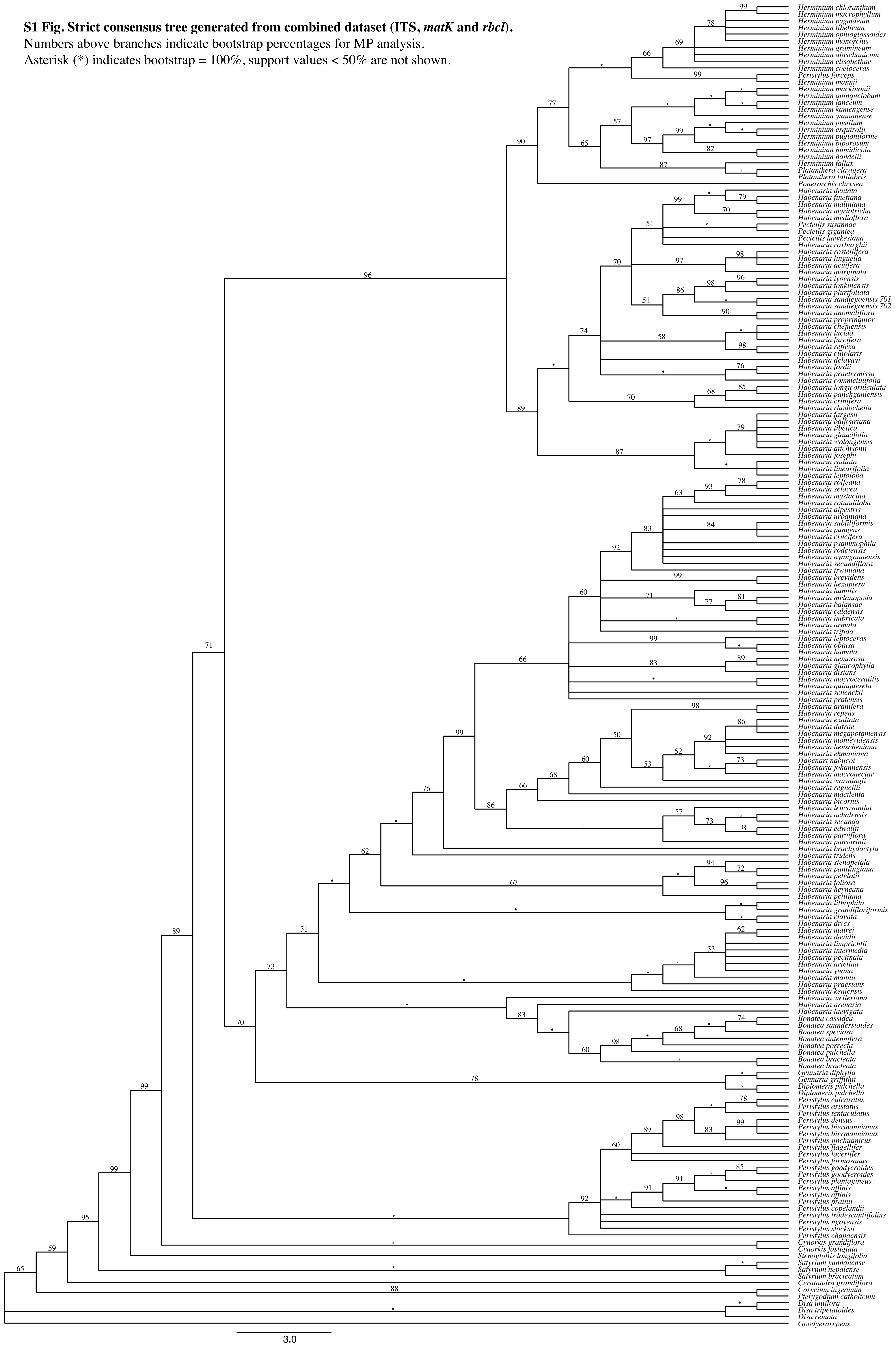

Supplement: S1 Fig — Numbers above branches indicate bootstrap percentages for MP analysis. Asterisk (*) indicates bootstrap = 100%, support values < 50% are not shown. (PDF) [file pone.0223355.s001.pdf]
