# Supplementary material for: Molecular phylogeny and morphology reveal a new epiphytic species of Habenaria (Orchidaceae; Orchideae; Orchidinae) from Nepal
Source: PLoS One. 2019 Oct 23;14(10):e0223355. doi: 10.1371/journal.pone.0223355 (PMC6808328; doi:10.1371/journal.pone.0223355)
Supplement: S1 Appendix — (*) Indicate sequences generated in this study. (DOC) [file pone.0223355.s006.doc]

**Appendix.** Taxa analyzed, voucher information, and GenBank accession numbers for the DNA sequences. (*) Indicate sequences generated in this study.

| Species name | Vouchers | ITS | *matK* | *rbcl* |
| --- | --- | --- | --- | --- |
| *Bonatea antennifera* | Truter JT419 (NU) | DQ522052 | DQ522081 | - |
| *Bonatea bracteata* | Truter JT687 (NU) | DQ522057 | DQ522084 | - |
| *Bonatea bracteata* | Truter JT262 (NU) | DQ522058 | DQ522084 | - |
| *Bonatea cassidea* | Truter JT965 (NU) | DQ522059 | DQ522085 | - |
| *Bonatea porrecta* | Truter s.n. (NU) | DQ522064 | DQ522088 | - |
| *Bonatea pulchella* | Truter s.n. (NU) | DQ522066 | DQ522089 | - |
| *Bonatea saundersioides* | Truter JT85 (NU) | DQ522068 | DQ522090 | KF589878 |
| *Bonatea speciosa* | Truter s.n. (NU), RL1158 | DQ522071 | EU214217 | EU213446 |
| *Ceratandra grandiflora* | Pauw & Liltved 49 (BOL) | EU687530 | EU687535 | - |
| *Corycium ingeanum* | Pauw A 11 (BOL) | EU301446 | EU301499 | - |
| *Cynorkis fastigiata* | T. Motley 2273 (NY) | MF944264 | MF350008 | AY381117 |
| *Cynorkis grandiflora* | Szlachetko s.n. | EF079186 | EF065584 | - |
| *Diplomeris pulchella* | Jin WT, Cui YQ 14354 | MF944269 | MF945434 | MF944869 |
| *Diplomeris pulchella* | Jin XH, Zhang L 11553 | MF944270 | MF945535 | MF944971 |
| *Disa remota* | Hitchcock 2057 | DQ414905 | DQ415048 | - |
| *Disa tripetaloides* | - | DQ414868 | DQ415011 | AF074151 |
| *Disa uniflora* | Harley s.n. | DQ414864 | DQ415007 | - |
| *Gennaria diphylla* | 109012 (SALA)  MW Chase O-886 | AY351380 | AY368383 | AY368341 |
| *Gennaria griffithii* | Jin10879 | JN696445 | JN696430 | JN696415 |
| Goodyera repens | KMC 1112 (NY) | FJ473327 | KT385636 | FJ571330 |
| *Habenaria achalensis* | JAN Batista 2506 (BHCB) | HM777526 | KJ021403 | - |
| *Habenaria acuifera* | Jin XH, Jin WT, Cui YQ 14403 | MF944278 | MF945429 | MF944865 |
| *Habenaria aitchisonii* | Jin XH, Jin WT, Xu SZ 13164 | KR350166 | KR350202 | KR350347 |
| *Habenaria alpestris* | JAN Batista 1576 (BHCB) | HM777655 | KJ021357 | - |
| *Habenaria anomaliflora* | Jin XH 11698 | MF944282 | MF945529 | MF944965 |
| *Habenaria aranifera* | JAN Batista 2472 | HM777626 | HM777819 | - |
| *Habenaria arenaria* | Salazar 6407 (K) | DQ522073 | HF560587 | - |
| *Habenaria arietina* | FLPH Tibet Expedition 13-1143 | MF944283 | MF945541 | MF944977 |
| *Habenaria armata* | JAN Batista 1297 (CEN) | HM777677 | KJ021367 | - |
| *Habenaria ayangannensis* | JAN Batista 1919 (BHCB) | HM777706 | KJ021360 | - |
| *Habenaria balansae* | JAN Batista 2336 (BHCB) | HM777683 | KJ021362 | - |
| *Habenaria balfouriana* | SET-ET 219 | MF944285 | MF945419 | MF944855 |
| *Habenaria bicornis* | LP Felix 10803 (EAN) | KF998087 | KX784170 | - |
| *Habenaria brachydactyla* | JAN Batista & P Proite 3109 | KX784176 | KX784171 | - |
| *Habenaria brevidens* | JAN Batista 2616 | HM777535 | HM777902 | - |
| *Habenaria caldensis* | JAN Batista 1798 (BHCB) | HM777644 | HM777882 | - |
| *Habenaria chejuensis* | - | KT338765 | KF262034 | KF296643 |
| *Habenaria ciliolaris* | Jin WT, Cui YQ 14352 | MF944286 | MF945424 | MF944860 |
| *Habenaria clavata* | Peter CP4185 (GRA) | DQ522074 | DQ522094 | - |
| *Habenaria commelinifolia* | YN-ET 907 | MF944288 | MF945456 | MF944890 |
| *Habenaria crinifera* | SBB-0768 | JN114582 | - | JN005479 |
| *Habenaria crucifera* | JAN Batista 1544 (CEN) | HM777574 | HM778013 | - |
| *Habenaria davidii* | Jin XH, Jin WT, Xu SZ 13200 | MF944289 | MF945536 | MF944972 |
| *Habenaria delavayi* | Jin XH, Jin WT, Xu SZ 13087 | MF944290 | MF945461 | MF944957 |
| | *Habenaria dentata* | | --- | | KFBG2126A, Jin XH 9411 | KY966605 | KY966891 | JN696423 |
| *Habenaria distans* | AC Amaral 18 (CEN) | HM777630 | HM777871 | - |
| *Habenaria dives* | Peter CP4104 (GRA) | DQ522075 | DQ522095 | - |
| *Habenaria dutrae* | M. Pedron 3 (ICN) | KJ021339 | KJ021387 | - |
| *Habenaria edwallii* | JAN Batista 1717 (BHCB) | HM777564 | KJ021402 | - |
| *Habenaria ekmaniana* | **-** | KJ021340 | KJ021383 | - |
| *Habenaria exaltata* | JAN Batista 2771 (BHCB) | HM777621 | HM777829 | - |
| *Habenaria fargesii* | Jin XH, Jin WT, Cui YQ 14644 | MF944292 | MF945407 | MF944843 |
| *Habenaria finetiana* | Jin XH, Jin WT, Cui YQ 14655 | MF944293 | MF945413 | MF944849 |
| *Habenaria foliosa* | SBB-0772 | JN114525 | JN004454 | JN005486 |
| *Habenaria fordii* | Jin WT, Cui YQ 14362 | MF944294 | MF945431 | MF944867 |
| *Habenaria furcifera* | Bhakta B. Raskoti 20147 | MF944296 | MF945473 | MF944907 |
| *Habenaria glaucifolia* | Jin XH, Jin WT, Xu SZ 13138 | MF944297 | MF945528 | MF944964 |
| *Habenaria glaucophylla var. glaucophylla* | JAN Batista 2428 (BHCB) | HM777632 | KJ021373 | - |
| *Habenaria grandifloriformis* | SBB-0755 | JN114536 | JN004463 | JN005500 |
| *Habenaria hamata* | C van den Berg 1266 (HUEFS) | HM777585 | KJ021371 | - |
| *Habenaria henscheniana* | JAN Batista 2802 (BHCB) | HM777623 | KJ021376 | - |
| *Habenaria hexaptera* | JAN Batista 2399 (BHCB) | HM777538 | HM777909 | - |
| *Habenaria heyneana* | SBB-0351 | JN114544 | JN004469 | JN005504 |
| *Habenaria humilis* | JAN Batista 1901 (BHCB) | HM777581 | KJ021364 | - |
| *Habenaria imbricata* | JAN Batista 1831 (BHCB) | HM777649 | KJ021368 | - |
| *Habenaria intermedia* | Bhakta B. Raskoti 201446 | MF944298 | MF945496 | MF944932 |
| *Habenaria irwaniana* | JAN Batista 249 (CEN) | HM777642 | KX784167 | - |
| *Habenaria iyoensis* | - | KT338766 | - | KF296647 |
| *Habenaria johannensis* | RC Mota 2777 (BHCB) | HM777609 | HM777841 | - |
| *Habenaria josephi* | Jin XH, Jin WT, Xu SZ 13261 | MF944299 | MF945540 | MF944963 |
| *Habenaria keniensis* | T274 | KX673554 | EF621492 | - |
| *Habenaria laevigata* | Johnson s.n. (NU) | DQ522076 | DQ522096 | - |
| *Habenaria leptoceras* | JAN Batista 2658 | HM777597 | HM777855 | - |
| *Habenaria leptoloba* | - | KY055535 | KY055545 | KY055556 |
| *Habenaria leucosantha* | JAN Batista 1604 (BHCB) | HM777569 | HM777790 | - |
| *Habenaria limprichtii* | Jin XH, Jin WT, Cui YQ 14654 | MF944301 | MF945411 | MF944851 |
| *Habenaria linearifolia* | - | KT338719 | KF262039 | KF296648 |
| *Habenaria linguella* | Jin XH, Jin WT, Cui YQ 14646 | MF944303 | MF945416 | MF944852 |
| *Habenaria lithophila* | Peter CP4085 (GRA) | DQ522078 | DQ522098 | - |
| *Habenaria longicorniculata* | SBB-0763 | JN114561 | - | JN005522 |
| *Habenaria lucida* | Hsu 1762 | MF944304 | MF945444 | MF944878 |
| *Habenaria macilenta* | JAN Batista 2393 (BHCB) | HM777606 | HM777812 | - |
| *Habenaria macroceratitis* | Chavez_s.n. (MEXU),  DB 1724 | HF560577 | EU214358 | - |
| *Habenaria macronectar* | JAN Batista 2519 (BHCB) | HM777614 | HM777833 | - |
| *Habenaria mairei* | Jin XH, Jin WT, Cui YQ 14678 | MF944305 | MF945414 | MF944850 |
| *Habenaria malintana* | Jin XH 9444 | MF944306 | MF945530 | MF944966 |
| *Habenaria mannii* | Salazar 6314 (YA) | HF560579 | HF560603 | - |
| *Habenaria marginata* | Bhakta B. Raskoti 201459 | MF944307 | MF945503 | MF944939 |
| *Habenaria medioflexa* | - | MF944308 | MF945439 | MF944874 |
| *Habenaria megapotamensis* | J. Klein 32 (UPCB) | KJ021347 | KJ021386 | - |
| *Habenaria melanopoda* | JAN Batista 2539 (BHCB) | HM777687 | HM777888 | - |
| *Habenaria montevidensis* | JAN Batista 2479 (BHCB) | HM777620 | HM777826 | - |
| *Habenaria myriotricha* | KFBG2959 | KY966606 | KY966892 | - |
| *Habenaria mystacina* | JAN Batista 1812 (BHCB) | HM777728 | HM777970 | - |
| *Habenaria nabucoi* | MO Pivari 549 (BHCB) | HM777611 | HM777840 | - |
| *Habenaria nemorosa* | JAN Batista 2567 (BHCB) | HM777634 | HM777872 | - |
| *Habenaria obtusa* | C van den Berg 1237 (HUEFS) | HM777588 | HM777862 | - |
| *Habenaria panchganiensis* | SBB-0752 | JN114567 | - | JN005526 |
| *Habenaria pansarinii* | JAN Batista 1926 (BHCB) | HM777520 | KX784168 | - |
| *Habenaria pantlingiana* | Bhakta B. Raskoti 201335 | MF944309 | MF945513 | MF944950 |
| *Habenaria parviflora* | JAN Batista 2470 (BHCB) | HM777561 | HM777800 | - |
| *Habenaria pectinata* | Gao XF, Zhu ZM, Ju WB, Jin WT 14714 | MF944310 | MF945539 | MF944975 |
| *Habenaria petelotii* | Jin WT 13562 | MF944311 | MF945526 | MF944962 |
| *Habenaria petitiana* | T276 | KX673555 | EF621493 | - |
| *Habenaria plurifoliata* | Jin XH 14581 | MF944312 | MF945459 | MF944893 |
| *Habenaria praestans* | Peter CP4186 (GRA) | DQ522079 | DQ522100 | - |
| *Habenaria praetermissa* | Luo YB 311 | MF944313 | MF945543 | MF944979 |
| *Habenaria pratensis* | JAN Batista 2686 (BHCB) | HM777546 | KJ021374 | - |
| *Habenaria proprinquior* | - | AF348035 | - | - |
| *Habenaria psammophila* | JAN Batista 1794 (BHCB) | HM777550 | HM778000 | - |
| *Habenaria pungens* | JAN Batista 2095 (BHCB) | HM777570 | HM778011 | - |
| *Habenaria quinqueseta* | Whitten 3324 (FLAS) | HF560581 | HF560605 | KJ773544 |
| *Habenaria radiata* | - | KT338720 | LC052787 | LC052788 |
| *Habenaria reflexa* | SET-ET 1073 | MF944314 | MF945537 | MF944973 |
| *Habenaria regnellii* | JAN Batista 2801 (BHCB) | HM777603 | KJ021392 | - |
| *Habenaria repens* | JAN Batista 2522 (BHCB | HM777627 | KJ021395 | AF074177 |
| *Habenaria rhodocheila* | Jin XH 8503 | KR350167 | KJ452799 | KJ451497 |
| *Habenaria rodeiensis* | RC Mota 2824 (BHCB) | HM777577 | HM777995 | - |
| *Habenaria rolfeana* | JAN Batista 2467 (BHCB) | HM777730 | HM777978 | - |
| *Habenaria rostellifera* | Luo YB 115 | MF944316 | MF945542 | MF944978 |
| *Habenaria rotundiloba* | JAN Batista 2684 (BHCB) | HM777717 | HM778017 | - |
| *Habenaria roxburghii* | SBB-0780 | JN005529 | JN004475 | JN005529 |
| *Habenaria sandiegoensis** *701* | Bhakta B. Raskoti 701 (KATH) | XXXXXX | XXXXXX | XXXXXX |
| *Habenaria sandiegoensis** *702* | Bhakta B. Raskoti 702 (KATH) | XXXXXX | XXXXXX | XXXXXX |
| *Habenaria schenckii* | JAN Batista 2882 (BHCB) | HM777580 | KJ021372 | - |
| *Habenaria secunda* | JAN Batista 2640 (BHCB) | HM777525 | HM777791 | - |
| *Habenaria secundiflora* | JAN Batista 2526 (BHCB) | HM777636 | KJ021358 | - |
| *Habenaria setacea* | JAN Batista 1417 (BHCB) | HM777731 | HM777979 | - |
| *Habenaria stenopetala* | SET-ET1277, PT 193 | MF944324 | MF945467 | MF944901 |
| *Habenaria subfiliformis* | JAN Batista 2022 (BHCB) | HM777573 | HM778008 | - |
| *Habenaria tibetica* | Jin XH, Jin WT, Xu SZ 13108 | MF944325 | MF945531 | MF944967 |
| *Habenaria tonkinensis* | - | MF944326 | MF945432 | MF944868 |
| *Habenaria tridens* | Truter JT182 (NU) | DQ522080 | DQ522101 | - |
| *Habenaria trifida* | JAN Batista 1783 (BHCB) | HM777672 | HM777917 | - |
| *Habenaria urbaniana* | JAN Batista 911 (BHCB) | HM777658 | HM777944 | - |
| *Habenaria warmingii* | JAN Batista 2584 (BHCB) | HM777617 | KJ021396 | - |
| *Habenaria weileriana* | Salazar 6310 (YA) | HF560585 | HF560609 | - |
| *Habenaria wolongensis* | SET-ET 944 | MF944327 | MF945532 | MF944958 |
| *Habenaria yuana* | - | KY055537 | KY055543 | - |
| *Herminium alaschanicum* | Jin XH, Jin W-T, Xu S. 13166 | KJ460100 | KJ452857 | KJ451555 |
| *Herminium biporosum* | sp0973 | KJ460042 | KJ452798 | KX527552 |
| *Herminium chloranthum* | STET 616 | KR350155 | KR350206 | KR350336 |
| *Herminium clavigerum* | Jin XH, Jin WT, Xu S. 13223 | KJ460078 | KJ452834 | KJ451532 |
| *Herminium coeloceras* | Jin XH, Jin WT, Xu SZ 13105 | KR350165 | KR350201 | KR350346 |
| *Herminium elisabethae* | FLPH Tibet Expedition 13-2104 | KR350161 | KR350197 | KR350342 |
| *Herminium esquirolii* | Jin XH, Jin WT, Xu SZ 13240 | KR350147 | KR350183 | KR350328 |
| *Herminium fallax* | Bhakta B. Raskoti 201333 | KR350150 | KR350186 | KR350331 |
| *Herminium gramineum* | Bhakta B. Raskoti 20133 | KR350151 | KR350187 | KR350332 |
| *Herminium handelii* | ST-1778 | KR350169 | KR350205 | KR350350 |
| *Herminium humidicola* | Jin XH, Jin WT, Cui YQ 14512 | KR350143 | KR350179 | KR350324 |
| *Herminium kamengense* | Bhakta B. Raskoti 20138 | KR350176 | KR350212 | KR350357 |
| *Herminium lanceum* | Bhakta B. Raskoti 20135 | KR350152 | KR350188 | KR350333 |
| *Herminium latilabre* | Jin XH, Jin WT, Xu S. 13222 | KJ460086 | KJ452842 | KJ451540 |
| *Herminium mackinonii* | Bhakta B. Raskoti 20131 | KR350148 | KR350184 | KR350329 |
| *Herminium macrophyllum* | Bhakta B. Raskoti 20137 | KR350175 | KR350211 | KR350356 |
| *Herminium mannii* | Jin XH 7983 | KR350162 | KR350198 | KR350343 |
| *Herminium monorchis* | FLPH Tibet Expedition 13-168 | KR350144 | KR350180 | KR350326 |
| *Herminium pugioniforme* | Jin XH, Jin WT, Xu S. 13264 | MF944336 | KJ452804 | KJ451502 |
| *Herminium pusillum* | Chung 11572 | KR350178 | KR350214 | KR350359 |
| *Herminium pygmaeum* | FLPH Tibet Expedition 13-2115 | KR350170 | KR350206 | KR350351 |
| *Herminium quinquelobum* | Bhakta B. Raskoti 20139 | KR350177 | KR350213 | KR350358 |
| *Herminium yunnanense* | Jin XH et Zhang Liang 11194 | KR350159 | KR350195 | KR350340 |
| *Hsenhsua chrysea* | STET 1292 | KJ460096 | KJ452812 | KJ451551 |
| *Pecteilis gigantea* | SBB-0840 | JN114651 | JN004549 | JN005610 |
| *Pecteilis hawkesiana* | - | MF944349 | MF945508 | MF944945 |
| *Pecteilis susannae* | Bhakta B. Raskoti 2014220 | MF944352 | MF945502 | MF944938 |
| *Peristylus affinis 1* | YN-ET 1008 | KR350163 | KR350199 | KR350344 |
| *Peristylus affinis 2* | Jin XH, Jin WT, Cui YQ 14623 | MF944353 | MF945406 | MF944842 |
| *Peristylus aristatus* | Bhakta B. Raskoti 201332 | MF944354 | MF945512 | MF944949 |
| *Peristylus biermannianus* | Jin XH 3132 | KR350294 | KJ452844 | KJ451538 |
| *Peristylus biermannianus* | Jin XH 8301 | KJ460088 | KJ452840 | KJ451542 |
| *Peristylus calcaratus* | Jin XH 7986 | MF944356 | MF945517 | MF944954. |
| *Peristylus chapaensis* | Jin XH 10084 | MF944357 | MF945520 | - |
| *Peristylus copelandii* | Hsu 7069 | MF944358 | MF945442 | - |
| *Peristylus densus* | Jin WT 11791 | MF944359 | MF945453 | MF944887 |
| *Peristylus flagellifer* | Jin10446 | JN696462 | MF945519 | MF944956 |
| *Peristylus forceps* | Jin9360 | JN696461 | KJ452789 | KJ451487 |
| *Peristylus formosanus* | - | KY055540 | KY055541 | KY055555 |
| *Peristylus goodyeroides 1* | Jin XH, Jin WT, Cui YQ 14643 | MF944362 | MF945410 | MF944848 |
| *Peristylus goodyeroides 2* | Jin XH, Jin WT, Cui YQ 14624 | MF944361 | MF945412 | MF944846 |
| *Peristylus jinchuanicus* | Jin XH, Jin WT, Cui YQ 14658 | MF944363 | MF945418 | MF944854 |
| *Peristylus lacertifer* | Jin XH 8507 | MF944366 | MF945477 | MF944912 |
| *Peristylus ngoyensis* | 324KM | - | AJ310054 | - |
| *Peristylus plantagineus* | SBB-0978 | JN114672 | JN004562 | JN005625 |
| *Peristylus prainii* | Jin XH 6958 | MF944334 | MF945518 | MF944955 |
| *Peristylus stocksii* | SBB-0831 | JN114578 | - | JN005536 |
| *Peristylus tentaculatus* | SET-ET 1281 | KJ460035 | KJ452787 | KJ451485 |
| *Peristylus tradescantiifolius* | Hsu 6779 | MF944371 | MF945441 | MF944876 |
| *Pterygodium catholicum* | MW Chase O-1130 | FJ469903 | FJ469845 | AY368346 |
| *Satyrium bracteatum* | T34, BB2110 | EF601480 | EF612540 | - |
| *Satyrium nepalense* | FLPH Tibet Expedition 13-1141 | JN114713 | EF612575 | MF944923 |
| *Satyrium yunnanense* | Jin XH, Jin WT, Cui YQ 14710 | EF601532 | MF945489 | MF944924 |
| *Stenoglottis longifolia* | MW Chase O-1136 | AF348065 | AY368387 | AY368349 |
